# Advanced care planning in the early phase of COVID-19: a rapid review of the practice and policy lessons learned

**DOI:** 10.3389/frhs.2023.1242413

**Published:** 2023-09-15

**Authors:** Sarah Younan, Magnolia Cardona, Ashlyn Sahay, Eileen Willis, Danielle Ni Chroinin

**Affiliations:** ^1^ Department of Geriatric Medicine, Liverpool Hospital, Sydney, NSW, Australia; ^2^ Institute for Evidence Based Healthcare, Bond University, Gold Coast, QLD, Australia; ^3^ School of Psychology, Faculty of Health and Behavioural Sciences, The University of Queensland, Brisbane, QLD, Australia; ^4^ School of Nursing, Midwifery and Social Sciences, Central Queensland University, Mackay, QLD, Australia; ^5^ South Western Sydney Clinical School, UNSW, Sydney, NSW, Australia

**Keywords:** COVID-19, advance care planning, policy, rapid review, barriers, enablers

## Abstract

**Background:**

The importance of advance care planning (ACP) has been highlighted by the advent of life-threatening COVID-19. Anecdotal evidence suggests changes in implementation of policies and procedures is needed to support uptake of ACPs. We investigated the barriers and enablers of ACP in the COVID-19 context and identify recommendations to facilitate ACP, to inform future policy and practice.

**Methods:**

We adopted the WHO recommendation of using rapid reviews for the production of actionable evidence for this study. We searched PUBMED from January 2020 to April 2021. All study designs including commentaries were included that focused on ACPs during COVID-19. Preprints/unpublished papers and Non-English language articles were excluded. Titles and abstracts were screened, full-texts were reviewed, and discrepancies resolved by discussion until consensus.

**Results:**

From amongst 343 papers screened, 123 underwent full-text review. In total, 74 papers were included, comprising commentaries (39) and primary research studies covering cohorts, reviews, case studies, and cross-sectional designs (35). The various study types and settings such as hospitals, outpatient services, aged care and community indicated widespread interest in accelerating ACP documentation to facilitate management decisions and care which is unwanted/not aligned with goals. Enablers of ACP included targeted public awareness, availability of telehealth, easy access to online tools and adopting person-centered approach, respectful of patient autonomy and values. The emerging barriers were uncertainty regarding clinical outcomes, cultural and communication difficulties, barriers associated with legal and ethical considerations, infection control restrictions, lack of time, and limited resources and support systems.

**Conclusion:**

The pandemic has provided opportunities for rapid implementation of ACP in creative ways to circumvent social distancing restrictions and high demand for health services. This review suggests the pandemic has provided some impetus to drive adaptable ACP conversations at individual, local, and international levels, affording an opportunity for longer term improvements in ACP practice and patient care. The enablers of ACP and the accelerated adoption evident here will hopefully continue to be part of everyday practice, with or without the pandemic.

## 1. Introduction

Advance care planning (ACP) has come into sharp focus, particularly given the severe impacts of COVID-19 on those who are older and frailer or who have high levels of comorbidity, with high fatality in these groups, as well as the notable pandemic-associated strains on resources (1–4).

ACP is “a process that supports adults at any age or stage of health in understanding and sharing their personal values, life goals, and preferences regarding future medical care”, aiming to ensure that medical care received is aligned with values and goals during serious and/or chronic illness (5).

Pre-pandemic, rates of ACP were not high. Population-based estimates of ACP prevalence in Australia hovered at 14% (6), while the US and UK had reported ACP engagement at ∼50%, and documentation at only 33% (7, 8). Rates of ACP pre-COVID-19 have been found to be lower in certain population subgroups, with lower rates, for example, amongst those of black, Asian and other “minority” American ethnicities, those identifying as LGBTQIA+ (Lesbian Gay Bisexual Transgender Queer and Intersex), as well as amongst homeless persons, incarcerated persons and those with limited health literacy (9–14). On the other hand, pre-existing disability and female sex may increase ACP uptake (15).

The relevance of ACP in the context of a global pandemic, featuring a life-threatening disease, and over-stretched healthcare resources, may seem self-evident. Yet factors which may positively or negatively impact on ACP, and options to support ACP in the context of COVID-19, have not been fully explored.

In this context, we conducted a rapid review of the literature, both to investigate barriers and enablers of ACP in the COVID-19 context, and to identify recommendations to facilitate ACP, in order to inform future policy and practice.

## 2. Methods

Given the rapidly evolving nature of the COVID-19 pandemic, we adopted the WHO recommendation of using rapid reviews for the production of actionable evidence (16).

### 2.1. Search strategy

We searched PUBMED for the period January 2020–April 2021, using MeSH terms “advance care planning”/“advance directive” plus “COVID-19”/“sars-CoV-2”. We screened reference lists of identified peer-reviewed articles with a focus on ACP during COVID-19.

#### 2.1.1. Inclusion and exclusion criteria

Inclusion criteria included papers which described or discussed ACP in the context of COVID-19, including rates (prevalence, incidence) of ACP, enablers and barriers, types of ACP, conduct of ACP, impact of the pandemic on ACP, and ACP-related outcomes. All study designs were eligible for inclusion, including commentaries and policy papers. Preprints, unpublished data and Non-English language articles were excluded.

### 2.2. Study selection

SY completed the literature search. MC and SY screened abstracts and reviewed all full texts. DNC, SY and MC reached consensus on all eligibility discrepancies. SY performed data extraction using a pre-defined table [author, publication year, country, study design, publication type, objectives, target population (if applicable), main conclusions]. MC cross-checked eligibility for inclusion. Given a rapid review, we did not formally conduct quality appraisal and risk-of-bias assessment.

### 2.3. Data synthesis and reporting

AS and EW reviewed data extraction tables and selected articles. Data were presented using simple descriptive statistics and tables complemented by a narrative synthesis.

## 3. Results

**Figure 1:**
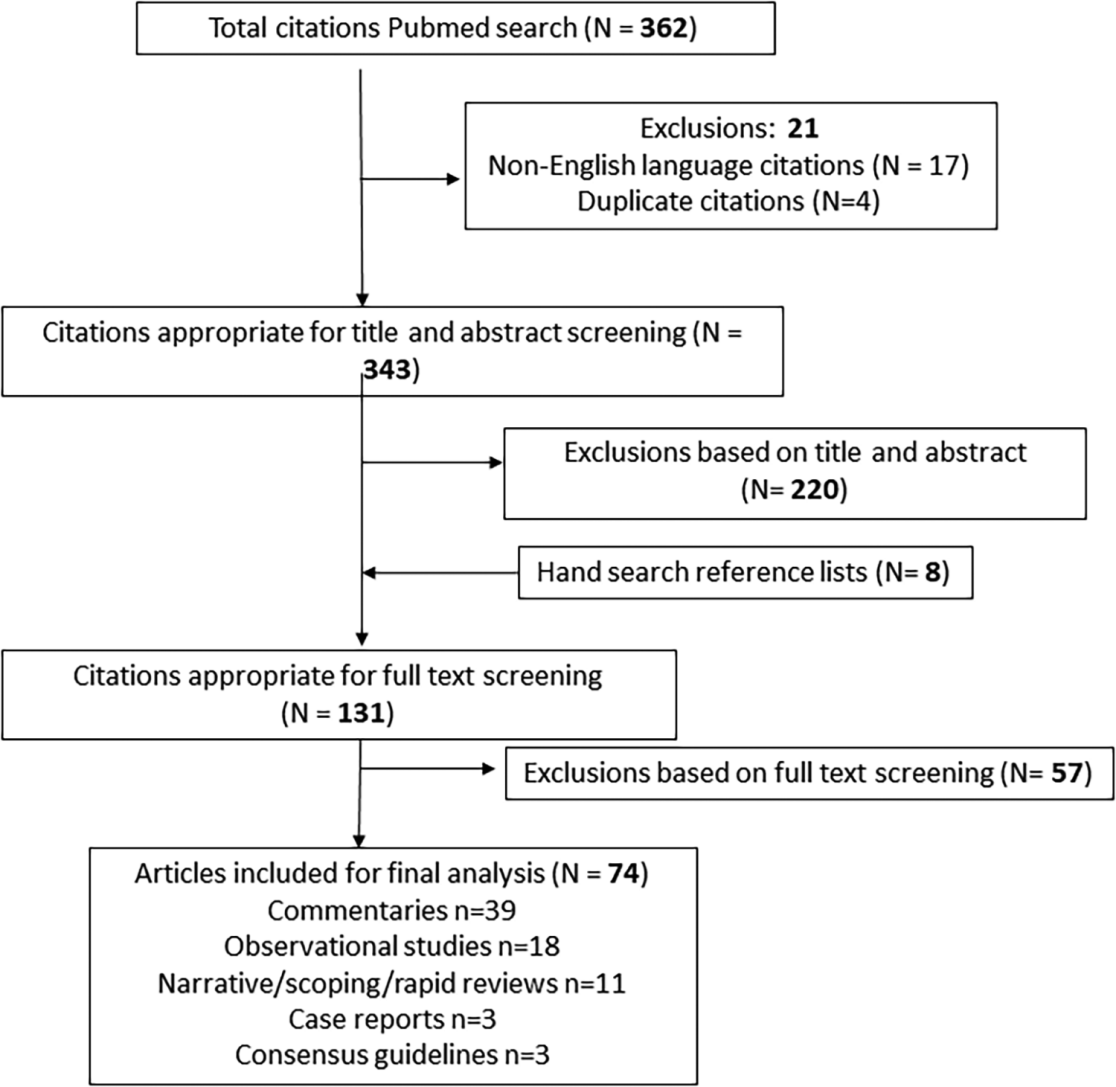
PRISMA diagram depicting outcomes of search and screening, full-text reviews, exclusions and final included studies (*N* = 74).

Overall, 343 studies were screened, with 123 full-text papers assessed for eligibility. After full-text screening, 74 were included, comprising commentaries (*N* = 39) or primary research studies (*N* = 35) (Figure 1).

Table 1 presents characteristics of included studies; Supplementary Material (online) gives further details. One-third (34%; *n* = 12/35) of the primary research studies focused on older adults, the remainder (66%; *n* = 23) targeting mixed age groups.

**Table 1:** Article details for primary studies and commentaries.

	Primary research (*n* = 35)	Commentaries (*n* = 39)
Country		
United States of America (USA)	23	26
United Kingdom (UK)	5	5
India	2	1
Taiwan	2	0
Japan	1	0
Netherlands	1	0
South Africa	1	1
Australia	0	2
Canada	0	1
Italy	0	1
New Zealand (NZ)	0	1
Singapore	0	1
Study design		
Commentaries	–	39
Cohort study—retrospective	9	
Cohort study—prospective	8	
Narrative review	9	
Consensus guidelines	3	
Rapid review	1	
Scoping review	1	
Case report	2	
Case series	1	
Cross-sectional study	1	
Study participants/focus		
Patients + Aged Care Clients	14	
Policy makers/Guidelines/Ethics	13	
General ACP users in the community	4	
Clinicians only	2	
Mixed: clinicians + patients	2	

There was significant overlap of key discussion points across commentaries and primary research studies, with themes and sub-themes identified as in Table 2. We explore these themes below.

**Table 2:** Emerging themes and Sub-themes.

Overarching themes	Sub-themes
1. Enablers of ACPs during COVID-19	1. Targeted public awareness and ACP engagement 2. Online platforms and Telehealth programs to facilitate AP discussions 3. Easy Access to online tools to guide ACP 4. Adopting a Person-centred approach and Respecting Patient Autonomy and Values
2. Barriers to ACP during COVID-19	1. Nature and novelty of the disease—acuity and uncertainty regarding clinical outcomes 2. Cultural and Communication difficulties for patients and clinicians, including lack of facility with IT 3. Barriers associated with Legal and Ethical Considerations/Concerns 4. Restrictions due to COVID infection control guidelines and procedures 5. Availability of time, resources and support systems
3. Recommendations for effective delivery of ACPs	1. Appropriate ACP design and structure 2. A collaborative approach using multidisciplinary teams 3. Increasing ACP education/literacy in the community 4. Upskilling and Training for clinicians 5. Addressing legal and ethical issues which may impede ACP

IT, information technology.

### 3.1. Enablers of ACPs during COVID-19

We identified several enablers of ACP from the included studies (Table 2).

#### 3.1.1. Targeted public awareness and ACP engagement

Three studies noted that COVID-19 had increased awareness and importance of completing ACD and legal documentation relating to dying (17, 18); one commentator reported a 3.5-fold increase in ACP requests leading to a surge in palliative/supportive care services (19). Several commentaries highlighted that the media focus on COVID-19, with daily mortality statistics, and stories of dying alone, brought ACP to the forefront of public attention and increased demand for ACPs (19–21).

Primary research studies indicated increasing emphasis on ACP for vulnerable groups, such as older adults (22–33), and high-risk groups with pre-existing life-limiting and/or chronic conditions, such as HIV (34), cancer or organ failure (23, 29, 35), cardiovascular/cerebrovascular disease (36) or severe mental illness (27). The evidence from these primary research studies was supported by perspectives in multiple commentaries (37–39).

Commentaries noted that in many aged and acute care settings, ACP was recommended for all patient (or resident) admissions (40, 41). Mooted options to increase uptake included sharing the ACP role with non-medical staff- social workers (42), nurses, or volunteers (19, 40, 43). Some primary research studies highlighted the value of opportunistic ACP discussions, in outpatient clinics (44, 45), prior to surgery (29) and/or at hospital admission.

#### 3.1.2. Online platforms and telehealth programs facilitating ACP

Studies highlighted that clinicians, patients and families used on-line platforms and telehealth technology to facilitate ACP. Several primary research studies and commentaries reported on uptake of outreach telehealth services and/or tool development (23, 37, 46–51).

Telehealth enabled socially/geographically isolated COVID-19 patientsand carers to maintain contact with healthcare professionals (HCPs), engage in ACP conversations, seek psychological support and symptom management (23, 50–52). Telehealth was also described as potentially offering benefits for those in financial difficulties (51), facilitating family e-meetings (50, 53) and enabling carers to engage in virtual farewells with dying patients (37, 46–49, 54, 55). Some authors noted that patients seemed relaxed in their home and more willing to engage in ACP discussions (45).

Telehealth communication reportedly increased satisfaction rates for clinicians and families (50). Telehealth also aided ACP upskilling and education for clinicians. While clinician ability to initiate ACPs may have been unchanged, one study noted junior doctors' confidence decreased and anxiety in undertaking ACP rose, possibly a Kruger Dunning effect, where increasing skill acquisition may increase awareness of incompetence (45).

#### 3.1.3. Easy access to online tools to guide ACP

Both primary research studies and commentaries noted accessible online/web-based ACP tools facilitated the development or adaption of COVID-specific end-of-life resources (17, 38, 56, 57). Existing tools such as Serious Illness Conversation guide (58) and online workshops (59, 60) were adapted to context. The available data suggested that online users were relatively young (mean 48 years) and largely female (67%) (18). A narrative review (57) also suggested that patient access to online health records might facilitate ACP documentation.

#### 3.1.4. Adopting a person-centred approach and respecting patient autonomy and values

Proponents of ACP argued that effective end-of-life care planning leads to treatment which was better aligned with patient wishes, and that ACP by its nature respects human rights; ACP might also be associated with reduced depression and anxiety among bereaved relatives, prevent unnecessary hospital admissions and reduce therisk of dying alone (21, 40, 49).

Several narrative reviews suggested ACP should and can embrace a person-centred approach to care, involving “effective” or “adequate, sympathetic” communication (26), and needs to be culturally appropriate (32). ACP was considered an opportunity to respectpatients’ autonomy and reflect their values, choices, and preferences (24, 26, 29, 32).

Several studies highlighted the importance of identifying a patient's surrogate decision-maker in emergency situations (24, 61). A narrative review cautioned that firstly, critically ill COVID-19 patients may be ill-disposed to make their wishes known, and should not be pressured to make decisions based on conserving resources, and secondly, physicians should not pre-emptively ration or pressurise older adults to reconsider their ACP or resuscitation preferences due resource availability (24).

### 3.2. Barriers to ACP during COVID-19

#### 3.2.1. Nature and novelty of the disease

Both primary research studies and commentaries identified that the rapid evolution of COVID-19 and uncertain clinical outcomes impeded ACP (46, 48, 62–67) A lack of prognostic clarity and varied treatment responses delayed ACP (66). Consensus guidelines, by the European Respiratory Society, reported that patients were too ill or anxious to participate in ACP, exacerbated by family absence. Rapid patient deterioration and/or clinical recovery added further complexity to timely discussions about ACPs and/or its currency (66).

#### 3.2.2. Cultural and communication difficulties

A narrative review reported that ACP language can be complex, which may impair communication and lead to inaccurate representation of patient goals (57). Three studies noted that comprehension of ACP forms is influenced by health and literacy levels (44, 61, 66); this finding was supported by several commentaries (15, 19, 20, 58, 68, 69).

Both primary research studies and commentaries highlighted that ACP uptake is lower in ethnic minorities or lower SES groups (12, 54, 57, 70). A rapid review identified an inverse relationship between ACP and socioeconomic status (SES) (32); with lack of trust in the healthcare system amongst people from certain population sub-groups a point echoed in the commentaries (32, 71). Furthermore, a narrative review (57) identified that some minority groups may be more likely to appoint a formal “Next of Kin”, but less often document ACP, which may increase burden for such substitute decision makers. Commentary papers highlighted that minority groups may lack familiarity with the health system, and that this may be exacerbated by limited access and communication/language barriers (12, 19, 20, 58, 69, 72). Two narrative reviews highlighted potential cultural taboos on discussing death (57, 61), while a commentary from Sub-Saharan Africa- where ACP is not recognized- noted a focus on efforts to “preserve” life (73). These views may change across generations or time, and may not reflect individual preferences even within specific sociocultural groups, highlighting the importance of adopting an individualised, patient-centred approach to ACP.

Evidence suggested that families, and not just patients, may also experience communication difficulties. Several commentators reported on family values, perceptions and understandings that may differ from those of clinicians (54, 64, 70, 71, 74, 75). Clinicians were reminded that familial conflict may compound these differences (52), and that individual preferences can change over time (76, 77). A US retrospective cohort study reported that families were more likely to change life support preferences when they attended in-person bed-side meetings, due to improved understanding of the patient's condition/prognosis (53). Commentators also noted that not all population groups have ready access to, or facility with, communication technologies such as telehealth, and that these may in turn be affected by language or SES (12, 70).

Clinicians also experience impediments to communication, for example, lack of confidence in communicating bad news (57) and difficulties in making speech heard when wearing personal protective equipment (PPE) (61). Clinicians may experience difficulties initiating ACP discussions with patients/families (57, 61) or hold concerns surrounding topic sensitivity and family grief (44, 61, 66). For example, clinicians may delay end-of-life conversations, reluctant to “take away” hope when prognosis is uncertain. A European Taskforce suggested a constant review process will maintain alignment of ACP to the patient's evolving condition (66). Three primary research studies, and several commentaries, recommended the need for improved clinician ACP training and access to palliative care education (57, 61, 66). Mitigating strategies proposed included ACP integration at all training levels (20), the integration of specific frameworks such as VitalTalk (63) or the use of other conversation guides to ensure equitable access and goal-aligned management (39, 40, 73), and ACP upskilling for allied health providers (67).

#### 3.2.3. Barriers associated with legal and ethical considerations

ACP laws and guidelines differ across countries and jurisdictions. Difficulties range from accessing lawyers (71), to differing legal terminology (15), to identifying who is entitled to initiate conversations. In some jurisdictions, only medical staff may initiate ACP conversations, with limited/no input from other multidisciplinary team members (67, 78). Mandated (over-)reliance on medical personnel may impede ACP- a large cohort study conducted in the US, found engaging social workers in ACP resulted in a 13% increase in patients nominating a medical POA (78). A Taiwanese study noted local legal obligations to have two witnesses and associated costs might impede ACP (79). A narrative review during the pandemic (26) identified seven documents that described core ACP issues to be discussed with nursing home residents: POA (Enduring Guardian equivalent), cardiopulmonary resuscitation (CPR), admission to hospital, intubation, non-invasive ventilation, fluids, antibiotics, etc. Supplementary Table S1 includes related resources, including a positionstatement by the American Geriatric Society (24).

A Canadian commentary argued that ACPs are less useful for COVID-19, lacking situational value, and difficulty fully informing individuals regarding treatment options, benefits and risks (65). They suggest patients should rather be asked about values, noting that ethical and legal issues are of serious concern during a pandemic. In particular, the ethics of blanket age- or residence-based protocols need to be considered when making advance care plans in the context of COVID. One commentary noted that some standardized protocols, e.g., in the UK, suggested that residential aged-care residents should not be admitted to hospitals (80). Such “one-size fits-all” approaches do not account for the uniqueness of individual presentations, prognosis and values (80). A US consensus guideline encouraged decision makers to focus on short-term outcomes and avoid age alone as a determinant of care. Other recommendations included avoiding ancillary criteria such as predicted long-term life expectancy (disadvantages older people), formation of triage committees and development of transparent resource-allocation strategies to facilitate appropriate ACP (24).

#### 3.2.4. Restrictions due to COVID infection control procedures

Almost all studies identified that COVID-related restrictions impacted peoples' ability to connect with clinicians and access ACP-related support. Infection control protocols governing the spread of COVID (social distancing, mask-wearing) impacted on communication between patients, families and HCPs. This, in turn, decreased the uptake of ACPs, as outlined in primary research studies (66, 79, 81) and commentaries (41, 58). For example, a UK based cohort study identified a decline (from 75.4% to 50.6%) in Do-Not-Attempt-Cardiopulmonary-Resuscitation due to restricted family visiting (81). The use of PPE and restricted visiting were hurdles to building trust required for effective ACP discussions (45, 66). These findings were ratified overwhelmingly by the commentaries (19, 46, 49, 52, 54, 62, 64, 69, 72, 75, 76).

Lack of interpersonal access increased family and patient distrust/suspicion towards HCPs (70), and/or the healthcare system (41, 82). Isolation, travel restrictions, and visitor limitations made shared decision-making extremely difficult. This was seen from descriptions of the potentially-dying patient deprived of in-person visits (41, 46, 48, 52, 58, 62, 64, 69), to reported feelings of being abandoned by medical staff (76). Stigma surrounding COVID, reinforced by the infection control protocols, exacerbated these difficulties and created barriers to ACP (73).

#### 3.2.5. Lack of time, resources and support systems

The enactment of ACP was identified as enabling fairer and more transparent resource allocation during periods of constraint, potentially reducing unnecessary life-sustaining treatments and streamlining the use of intensive care resources (1, 25, 35, 36). This was seen to alleviate the need for healthcare rationing, by avoiding aggressive treatment for those who do not want it, engaging patients in shared-decision making and avoiding blanket decisions based on age or comorbidity. Four commentaries noted that ACP also potentially reduced clinician exposure to avoidable infection and risk (e.g., with CPR), and assisted in planning for surges in healthcare demand (19, 21, 49, 77).

Nonetheless, the process of formulating ACP was recognised to be time intensive. Several primary research studies highlighted that clinicians are time poor, with little time for lengthy discussions or to develop clinician-patient relationships and trust (26, 35, 36, 57, 61). Numerous commentaries supported this finding (46, 58, 64, 67, 69, 70, 76). Commentators noted a lack of appropriate policies highlighting ACP's importance, a failure to prioritize it (46, 49, 54, 62, 63), and either poor or overly-cumbersome documentation, as well as failure to remunerate clinicians appropriately for time invested e.g., with dedicated billing codes (46).

Despite these barriers, requests for ACP have reportedly increased since the pandemic across varied settings: aged-care, terminal neurological patients, and prisons (17, 32, 38–41, 44, 82), with goals of care discussions ranging from 36% (53) to a 3.5-fold increase from baseline (19).

##### 3.2.5.1. Recommendations for effective ACP delivery

The available evidence highlighted a number of recommendations to facilitate effective ACP delivery (Table 2).

#### 3.3.1. Appropriate ACP design and structure

Various commentators suggested a need to develop or adapt tools specifically to the COVID context (17, 38), and, given the emotional impact of the diagnosis, to deliver small chunks of information at a time (15, 42, 63, 75). Three editorials suggested that current ACP tools are targeted towards non-communicable chronic illnesses rather than the COVID experience (52, 64, 70), and thus may require adaptation to best serve the COVID-19 context. Other authors provided specific ACP tools, approaches or frameworks to guide clinicians and facilitate standardization (58, 83, 84). Similar suggestions were made in primary research studies, but specific recommendations for design and structure of ACPs were limited.

#### 3.3.2. A collaborative approach

Multidisciplinary collaboration was encouraged, guided by specialists trained in ACP or palliative care (22, 43, 82), although it was noted that specialist teams are not universally available (39). Other suggestions included that non-medical team-members should assist with ACP, e.g., nurses and social workers (40, 67), or spiritual carers (19). Frameworks and tools were seen to address such concerns and to improve clinician confidence (39, 40, 42). A commentary by Ballantyne et al. (64) suggested that interpersonal barriers of social distancing could be overcome by increasing pre-illness ACP discussions.

#### 3.3.3. Increasing community ACP education/literacy

The importance of promoting and increasing ACP education and literacy for the wider community was frequently recognised (20, 42, 61, 66, 85), including relationship-building with community, ethnic and religious groups (19, 68).

One commentary suggested that hospital-based policies, jurisdictional legislation and community campaigns needed to accompany the broadening ACP education for HCPs (67). Death education/literacy may be best embedded across all health curriculum levels (20). Other options proposed included templates and tools to facilitate ACPs, videos and telehealth to facilitate remote ACP, and creation of an integrated web-based system linked to electronic medical records (eMR) to facilitate ACP in the community (18, 48, 60, 61, 85).

Several other studies indicated a need to promote ACP literacy, but specific strategies were lacking (28, 56, 79, 86).

#### 3.3.4. Upskilling and training for clinicians

Upskilling clinicians to improve ACP confidence was a recurring theme, highlighted by at least two narrative reviews (57, 87) and a consensus guideline (66). Proposed teaching strategies were varied, e.g., role-play, interactive multimedia, virtual training, online communication, and telephone debriefing (45, 85). One review suggested that training HCPs in assisted living communities could help communication across services (31).

#### 3.3.5. Addressing legal and ethical issues which may impede ACP

Legal and ethical issues featured predominantly in commentaries. Suggestions included temporarily pausing stricter legal requirements for ACP during COVID (15). Several studies addressed resource utilization and most studies argued that ACP should be prioritized to reduce unwanted intensive/life-sustaining treatment in a stretched healthcare system (88). Clinician concerns regarding the ethics of CPR in COVID-19 patients were also noted (47). Nevertheless, one institution highlighted that resource availability and patient/family goals of care need to be balanced when making resuscitation decisions (74).

## 4. Discussion

This rapid review of ACP implementation in the initial phase of the pandemic (until April 2021) suggests a high level of worldwide interest since COVID emerged. ACP may benefit overwhelmed healthcare systems, and decision-making for patients, caregivers and clinicians, and improve bereavement experiences. A sense of urgency and eagerness to make recommendations was apparent, often without supporting high-quality evidence, as evident from the large proportion of commentaries (39), consensus guidelines (3) and case series/reports (3) included in this review. Most publications were from the USA and UK ─ severely affected countries, but also nations which are better resourced to produce and promote research, compared, for example, to economically developing countries, from which data were scarce. Settings spanning acute hospitals, community, aged care and outpatient services showcased the breadth of the locations affected, and the need for ACP in a diverse range of contexts.

We were not surprised by the focus on barriers and enablers of ACP during the pandemic. Many of these may be familiar to clinicians and health services managers in their own place of work. Some of the enablers were specific to the nature of a pandemic, such as the improved public awareness in the context of a life-threatening communicable condition. Others may seem intuitive, e.g., embracing a patient-centred approach to care (89), but are not always evident in practice. The variety of proposed solutions was reassuring in terms of timeliness and feasibility, with standardization, collaboration, education and ethical-legal considerations underpinning implementation into practice.

Two main lessons surfaced from this rapid review. First, the prevalence and clarity of goals of care discussions were often touched on as a priority issue for patient management, prevention of unwanted (non-goal-aligned) care, or transfer decisions, across the range of study types (31, 34–36, 50, 53, 57, 82, 90). Next, frequent references to online tools or resources (18, 56, 58, 83, 87) and virtual learning for clinicians and families including online family meetings (45, 50, 53, 57, 59, 60, 66), reflect rapid embracing of innovative approaches to facilitate ACP completion and circumvent face-to-face interactions. Both are reassuring findings which indicate that patient/family wishes on end-of-life care were not neglected in the chaos of the unanticipated demand for health services, although frequency and equity of access were not always optimal (53, 57), and absence of communication was perceived to lead to complex bereavement (61, 91).

A poignant aspect of COVID-19 was the vision of people dying in alone in hospital, unable to be with family and suffering respiratory discomfort. For families, this led to a complicated grief experience characterized by survivor guilt, anger and distress, while the true impact on the patients themselves who did not survive has not been well-captured. An obvious mitigating strategy in such circumstances was to increase ACP so all stakeholders were prepared, patients received the care they desired and symptoms were managed in line with individualised goals (40, 68, 76, 91, 92).

This review also identified several recommendations which were mooted to facilitate effective ACP: appropriate ACP design and structure, adoption of a collaborative, multidisciplinary approach to ACP, increasing public and community education and literacy in relation to ACP, upskilling of clinicians, and addressing legal and ethical issues which may be impede ACP. While existing frameworks may provide some foundation, these may require adaptation, not only for the context of the pandemic, but also to account for local population needs and resources. Related to this, embracing technological advances and online ACP platforms must be balanced against patient preferences and acceptability, and cognizance of potential consumer concerns, such as those identified, for example, regarding use of personalized health records, aspects like ease of use, usefulness and security risk (93).

The ethics of ACP, and the need to balance principles of autonomy, beneficence, maleficence and justice (94), are not unique to the pandemic context, but particular aspects have come to the fore in the context of resource strains (4). Furthermore, ACP is now, in contrast to its former documented-directive focused origins, acknowledged to be a largely communicative process (94). This brings with it rich opportunities to maintain a dynamic conversation with patients and their loved ones as values and preferences, and physical status and related prognosis, change over time.

### 4.1. Implications for practice

Our pandemic-related experience has pushed us to re-evaluate existing healthcare policies and practices, overcome new and longstanding barriers, and embrace new solutions, in ACP as in other areas of practice. Recommendations to improve ACP delivery were highlighted by this review, as above. Our findings suggests the pandemic has provided some impetus to drive adaptable ACP conversations at individual, local, and international levels. It has provided capacity, opportunity and motivation to review and enhance our existing ACP practices, the three ingrediaents for successful behavioral change (95). Hopefully the changes instituted and lessons learned have afforded an opportunity for longer term improvements in ACP practice and care that is respectful of, and responsive to, the values and needs of the individual patients we meet every day. Institution and maintenance of any such changes should be combined with assessment in order to identify ongoing gaps and opportunities for improvement.

### 4.2. Limitations of the review

Given the nature of a rapid review, we only used limited number of search terms which were specific to our objective, confined to searching English language publications in the early stages of the pandemic and from a single (but the largest) database, anticipated to contain the majority of relevant articles. Half of the publications reflected perception, early reactions and proposed solutions and were not interventions or evaluations of policies or practices. Our intention was to illustrate all perspectives given the uniqueness of this serious pandemic experience, and thus we included a broad range of literature, but also highlighted the empiric evidence from primary research studies separate to that from commentaries. A future review of subsequent publications in the late stages of the pandemic may report similar or different findings, and it would be interesting to compare our results with emerging lessons after longer exposure to the global threat, as well as in the non-English based literature. Findings from nations which were under-represented in the current search ad review would also be of interest.

## 5. Conclusion

Despite high demand for healthcare services, the pandemic provided opportunities for rapid implementation of ACP. Both barriers and enablers of ACP influence its uptake, and these need to be considered at all levels of healthcare planning- local, national and international- if ACP is to achieve wider reach. Evidence suggests clinicians, patients and families support the recent cultural shift that fosters positive ACP uptake; this may contribute to reduced overtreatment near end of life and improve patient and family experiences of severe illness and care (96). Ongoing evaluation of policy changes, effectiveness, acceptability and patient and family satisfaction of ACP implementation are warranted in order to guide best practice moving forward.
