# Supplementary material for: Advanced care planning in the early phase of COVID-19: a rapid review of the practice and policy lessons learned
Source: Front Health Serv. 2023 Sep 15;3:1242413. doi: 10.3389/frhs.2023.1242413 (PMC10541151; doi:10.3389/frhs.2023.1242413)
Supplement: Supplementary file 1 [file Table1.docx]

Supplementary Material

**Advanced Care Planning (ACP) in the early phase of COVID-19: A rapid review of the practice and policy lessons learned.**

*Sarah Younan, Magnolia Cardona, Ashlyn Sahay, Eileen Willis, Danielle Ni Chroinin^*^.*

*** Correspondence:**Corresponding Author
[Danielle.Nichroinin@health.nsw.gov.au](mailto:Danielle.Nichroinin@health.nsw.gov.au)

**Supplementary Data:**

Summary of Publications and Resources for Cohort Studies, Consensus Guidelines, Narrative Reviews, Cross-Sectional, Case Reports & Commentaries:

**Supplementary Table 1: COHORT STUDIES**

**Supplementary Table 2: GUIDELINES**

**Supplementary Table 3: REVIEWS**

**Supplementary Table 1: COHORT STUDIES**

| **Authors,  Year,  Journal** | **Study design:**  **Cohorts /country** | **Population and setting** | **Objectives** | **Resources (when available)** | **Main findings & comments** |
| --- | --- | --- | --- | --- | --- |
| Auriemma CL, Halpern SD, Asch JM, Van Der Tuyn M, Asch DA 2020 JAMA Network | Cohort study - prospective  USA | Users of ourcarewishes.org - a free online tool to guide ACP | To quantify changes to patient completion of advance directives and expressed preferences during COVID-19 pandemic by monitoring users of a web-based advance care planning (ACP) platform from January 2019 to April 2020 | Users of ourcarewishes.org - a free online tool to guide ACP  OurCareWishes.org <https://www.ourcarewishes.org/> | During the pre-COVID period there were 424 users, with a median (IQR) of 26 (22-30) monthly new users, 5 (3-6) monthly returning users, and 31 (28-34) monthly total users. During the COVID-19 period, there were a median (IQRs) of 133 (71-207) monthly new users, 21 (11-39) monthly returning users, and 154 (82-246) monthly total users. This represents a 4.9-fold increase in monthly users in the COVID-19 period compared with the pre–COVID-19 period.  The reasons for increased demand for ACP documentation could be explained by a sense of increased importance of ACP during COVID-19, the push for clinicians to promote ACP or new motivation for people wanting to complete ACP but who have never done so. |
| Back AL, Grant MS, McCabe PJ 2021 J Palliat Med | Cohort study – prospective  USA | Two focus groups conducted before and during COVID-19 pandemic | To use focus groups to understand public perceptions of serious illness care that can be used to design for greater public engagement | Two focus groups conducted before and during COVID-19 pandemic | 82 people from the general population participated. They were aged 50-80 years, 50% male, 30% from diverse backgrounds.  There were three main observations;  1. Misunderstanding about ACP, palliative care and hospice care is widespread.  2. COVID-19 evokes its own brand of confusion and ambivalence - all were reluctant to go to hospital if they had COVID-19 due to not wanting to die alone 3. Distrust of the health care system is the new normal. |
| Berning MJ, Palmer E, Tsai T, et al 2021 J Am Geriatr Soc | Cohort study – prospective  USA | Residents at 2 nursing homes in Boston, USA with activated healthcare proxies who lacked DNH directives as of April 13, 2020. Trained HCW contacted proxies to conduct COVID-19 focused ACP discussions | To describe a systematic approach to address ACP during a COVID-19 outbreak and its impact on the incidence of new Do-Not-Hospitalise (DNH) directives among nursing home residents. | Supplementary discussion guide  <https://agsjournals.onlinelibrary.wiley.com/action/downloadSupplement?doi=10.1111%2Fjgs.17051&file=jgs17051-sup-0001-supinfo.pdf> | At baseline, 315/518 (54%) residents did not have DNH directives - 70% were female, mean age 87 (+/-9) years, 33% had moderate-severe cognitive impairment, 24% had a Do-not-resuscitate directive. Following the intervention, 124 (39%) of the 315 residents acquired new DNH directives - 52% went on to be diagnosed with COVID-19 and 23% died during follow-up. Among all residents that ended up with DNH directives, only 3 (2%) were hospitalised.  Enablers to implement this ACP initiative included a robust electronic Medical Record (eMR), multi-disciplinary team structure and strong stakeholder involvement. |
| Kuntz JG, Kavalieratos D, Esper GJ, et al  2020  Journal of pain and symptom management | Cohort study – prospective  USA | The inpatient team at Emory Palliative Care Centre, Georgia USA, conducted family meetings via Zoom. | To implement and evaluate the use of telemedicine on the family meeting | E-family meeting procedure guide to use on Zoom provided | 67 e-family meetings were conducted for 63 patients, 70% of whom were COVID-19 positive and 59% of whom were intubated. The main reasons for family meetings were to provide support (90%), provide clinical information (38%) and clarify goals of care (35%). The average clinician rating of e-family meetings was 3.18/5 (SD 0.96). There was a high degree of satisfaction among clinicians and families using e-family meetings. |
| Osterman CK, Triglianos T, Winzelberg GS, et al  2021  Support Care Cancer | Cohort study – prospective  USA | Haematology and Oncology patients in North Carolina, USA | To risk-stratify oncology patients to identify those at high risk for acute healthcare utilization during COVID-19 pandemic and pilot implementation of a telephone-based outreach for high-risk patients. | Patient outreach documentation template of standardized questions and recommended interventions for identified patient need  <https://link.springer.com/article/10.1007/s00520-020-05744-y> | 1697 patients were risk-stratified. A Health Composite Score (HCS) is predictive of 90-day hospitalisation. The Cancer-specific risk (CRS) was also calculated. Both were used for risk stratification to assess likelihood of acute healthcare utilisation. By HCS and CSR, 17% and 22% of patients were high risk respectively.  The outreach telehealth program focused on questions and interventions around symptoms, medications, coping, resources, ACP, and cancer care. In the first month 286 patients were called and 245 (86%) were successfully contacted. Commonly identified patient issues were financial difficulties (17%), uncontrolled symptoms (15%), and interest in ACP (13%). Overall, 33% of patients were referred for additional services.  The pandemic has forced rapid changes to healthcare delivery, including increased utilization of telemedicine, which may introduce new barriers to care and new challenges to systematically addressing patient concerns, in turn increasing the risk for acute healthcare utilization. This telehealth intervention identified a high burden of unmet medical and psychosocial needs in oncology patients during the COVID-19 pandemic. |
| Mills S, Cioletti A, Gingell G, et al  2021  J Pain Symptom Manage | Cohort study – prospective  USA | 48 residents across a health centre and outpatient clinic in Texas, USA. During April and May 2020, a new mandatory ACP curriculum was implemented. | To create a virtual ACP curriculum to improve residents' confidence and communication skills around ACP in the outpatient setting. | Supplementary question guide for patients at  <https://www.jpsmjournal.com/article/S0885-3924(21)00273-6/fulltext#relatedArticles> | The COVID-19 pandemic highlights the urgency of teaching ACP to residents. The new ACP curriculum included pre-readings, virtual training and peer role-play. Residents then engaged 5 patients in ACP phone conversations, followed by virtual group debrief. Residents' confidence in conducting ACP conversations before and after the intervention showed no significant change except for discussing advance directives, where residents' confidence decreased significantly post training. This seems paradoxical but is consistent with the Kruger Dunning effect which suggests that increasing skill enhances awareness of previous lack of competence.  Four major themes were identified from qualitative data;  (1) Contrasting outpatient and inpatient settings - inpatient ACP is urgent, immediate and late whereas outpatient ACP provides more time and longitudinal relationships, however residents are less familiar with the outpatient setting.  (2) ACP conversations provoke anxiety in residents.  (3) The benefits and challenges of telehealth - patients are more relaxed in the comfort of their home and more willing to have these discussions, however language barriers and the inability to read facial and physical cues are barriers to ACP via telehealth.  (4) Positive learner experience - residents gained valuable experience through the program. |
| Smith GM, Hui FA, Bleymaier CR, Bragg AR, Harman SM  2020  Journal of Pain & Symptom Management | Cohort study – prospective  USA | Two-part virtual ACP workshop developed at Stanford Health Care, California | To describe the implementation and evaluation of a novel, public-facing, two-part virtual ACP workshop | Stanford Palliative Care  <https://med.stanford.edu/palliative-care/COVID19.html>  Two-part virtual ACP workshop developed at Stanford Health Care, California | 570 accounts registered for Part 1 workshop and 413 accounts logged in to participate. 91 accounts then participated in Part 2. 98 and 39 participants completed the post-session survey after Part 1 and Part 2 respectively. On a five-point Likert scale, the mean likelihood to recommend score for Part 1 was 4.0 (SD 1.4) and 4.2 (SD 1.4) for Part 2. The mean overall ACP engagement score after Part 1 was 4.0 (SD 0.8) and 3.8 (SD 0.7) after Part 2. The online workshop was a successful way to engage the public in ACP. |
| Singh S, Herrmann K, Cyriacks W, et al  2021  J Pain Symptom Manage | Cohort study – prospective  USA | A large tertiary referral hospital in Denver, Colorado, USA. Between April and June 2020, all hospitalised patients were screened for a documented MDPOA in the last 12 months. | To assess the impact of a social work and care management intervention on the proportion of admitted patients each week with a Medical Durable Power of Attorney (MDPOA). |  | All hospitalised patients were screened for a documented MDPOA in the last 12 months and those without MDPOA in eMR were referred for the social work intervention. Prior to the intervention, on average 30.1% of patients had MDPOA forms documented in eMR. Post-intervention, the rate of MDPOA completion was 42.8% on average. The number of social work referrals overwhelmed the capacity of the department and resulted in the need to target high-risk patients only (including those with COVID, the elderly, oncology and trauma patients). |
| Ye P, Fry L, Champion JD 2021 Journal of the American Medical Directors Association | Cohort study – retrospective  USA | Nursing home residents or their surrogate decision makers in 15 nursing homes in Southwestern USA. | To describe the care preference changes among nursing home residents receiving proactive ACP conversations from health care practitioners during COVID-19 pandemic |  | 963 residents were included in the study. Most residents were women (62.1%) and age ranged from 22 to 105 years (mean = 78 years). At least one ACP conversation was had between the resident and health care worker between April and May 2020. Nursing home residents and their surrogates do change their care preferences significantly after ACP conversations.  Residents' chosen code status BEFORE Vs AFTER ACP conversation: - full code: 37.5% vs 28.3% - Out of Hospital Do Not Resuscitate: 62.5% vs 71.7% - Do not hospitalise 19.5% vs 47.2%  This study demonstrated that nursing home residents and their surrogates do change their care preferences significantly during the current COVID-19 pandemic. |
| Burke RV, Rome R, Constanza K, et al  2020  Palliat Med Rep | Cohort study – retrospective  USA | COVID-19 patients admitted to ICU in a hospital in New Orleans, USA, between March 29 and May 1 2020. | To pilot a team-based structured protocol to categorize severity of COVID-19 ICU admissions and collaborate with the palliative care team to assess physical, spiritual, and psychosocial needs | Provides text for Palliative Care Social Work Phone Contact prompts | All (n=19) COVID patients in ICU received a palliative care consult. 74% of consults resulted in completion of an advanced care directive. 58% of consults were completed using video. 100% of goals-of-care conversations occurred in person.The team-based approach decreased the facility burden of caring for seriously ill COVID-19 patients |
| Coleman JJ, Botkai A, Marson EJ, et al 2020 Resuscitation | Cohort study – retrospective  UK | A large urban teaching hospital in UK. Data extracted from eMR for DNACPR forms completed between 1 January 2017 and 30 April 2020. | To analyse eMR documentation on treatment limitation and Do-Not-Attempt-Cardiopulmonary-Resuscitation (DNACPR) decisions comparing uptake, decisions and answers to different sections of the form before and during the COVID-19 situation | Resuscitation Council  <https://www.resus.org.uk/covid-19-resources> | Forms completed prior to 1 March 2020 were considered "pre-COVID". Forms completed between 1 March and 30 April 2020 were considered "during COVID". 16,007 forms were identified during the study period, including 1844 forms (11.5%) during COVID. Patients completing DNACPR forms during COVID were younger, less likely to have co-morbidities and less likely to have a referral to the palliative care team. During COVID, significantly more patients had documents stating CPR would be appropriate (23.8% vs 9.05%, p<0.001) and for full active treatment (30.5% vs 26.1%, p=0.028). There was a significant decrease in DNACPR decisions being discussed with relatives - dropping from 75.4% to 50.6% (p < 0.001), likely due to visitor restrictions. This data suggests that there was a shift during the COVID-19 pandemic from undertaking ACP conversations and DNACPR decision only in critically ill patients to a much wider group of inpatients. |
| Funk DC, Moss AH, Speis A 2020 Journal of Pain & Symptom Management | Cohort study - retrospective  USA | The West Virginia Centre for End of Life Care, USA | To describe the impact COVID-19 had on ACP based on changes in the calls to the West Virginia Center for End-of-Life Care and in the volume and types of documents requested from and submitted to the center and its e-Directive Registry between January to June 2020 | Center to Advance Palliative Care. ‘‘Fast five:’’ Legal issues in advance care planning during COVID-19. CAPC.org <https://www.capc.org/covid-19/communication/advance-care-planning/> | Calls to the centre during this six month period related to four main topics;  1. Confirmation of documents in the registry  2. Urgent desire to initiate advance care planning  3. Request for temporary rescindment of treatment-limiting forms  4. Patient-specific questions about how to honour patients’ wishes in advance directives and medical orders in light of their COVID-19 status. The number of advance directive forms distributed in this time period was the highest in the last five years. There was a 77.18% increase in Do-Not-Resuscitate cards received in the first six months of 2020. |
| Lin MH, Hsu JL, Chen TJ, Hwang SJ  2021  J Chin Med Assoc | Cohort study – retrospective  Taiwan | 15 veterans’ hospitals in Taiwan providing outpatient ACP services from 2019 to June 2020. | To investigate the effect of COVID-19 on the outpatient ACP services provided by veterans hospitals in Taiwan |  | 2493 individuals received ACP services between April 2019 and June 2020. ACP services declined from 206.2 ± 29.2 declarants per month (before COVID-19) to 106.2 ± 30.8 declarants per month (during COVID-19). During the pandemic, those who received ACP services were younger, more likely to be health-care workers, and more likely to pay for the ACP services. Overall, there was significantly less ACP completed during COVID-19 period. This was likely due to the public complying with government restrictions, staying home and cancelling non-necessary outpatient appointments to reduce risk of transmission.  Obstacles hindering people from seeking, receiving, or offering ACP services included;  - the general public’s lack of knowledge and understanding pertaining to ACP  - hospitals lacking the mechanisms needed to provide ACP services  - shortages in private space in wards or outpatient environments  - difficulty finding two witnesses to complete the document-signing process  - medical staff did not possess sufficient ACP knowledge and avoided talking about death-related matters, were subjected to the clinical pressure of not having enough time, and were sceptical whether the legal effects of the ACP would affect declarants’ willingness to engage. |
| Piscitello GM, Fukushima CM, Saulitis AK, Tian KT, Hwang J, Gupta S, et al  2021  Am J Hosp Palliat Care | Cohort study – retrospective  USA | Patients admitted to ICU at an urban academic hospital in Southwest USA between March to June 2020, who lacked decision-making capacity and received a referral for a video meeting | To evaluate the quantity of family meetings by telephone, video and in-person during COVID-19 pandemic |  | 61 patients met the study's inclusion criteria and 93% had these had COVID-19. 61% patients died during the hospital admission. 7% of patients had a documented Power of Attorney. In total, 650 family meetings were documented for these 61 patients. Only 36% of these family meetings discussed patient goals of care. Most family meetings occurred by phone (59%), whereas 8% were conducted by video, 11% were conducted in-person and 22% unknown. The majority of in-person meetings (57%) discussed goals of care and a change in goals of care was more likely to occur for in-person meetings because families can better understand patient’s condition when at the bedside. On average, patients had a documented family meeting every 2 days during their admission which is less than other studies (pre-COVID) which showed family meetings every 1.2 days per patient in ICU. Barriers to video conferencing including access, elderly family members and cost. |
| Portz JD, Brungardt A, Shanbhag P, Staton EW, Bose-Brill S, Lin CT, et al  2020  Journal of medical internet research | Cohort study - retrospective  USA | Web-based ACP tool available through the University of Colorado Health patient portal- data collected between January and May 2020 | To determine the rates of use of a web-based ACP tool through an electronic patient portal before and during COVID-19 pandemic | Advance Care Planning. National Hospice and Palliative Care Organization.   URL: <https://www.nhpco.org/patients-and-caregivers/advance-care-planning/>  Advance Care Planning: Healthcare Directives. National Institute on Aging.   URL: <https://www.nia.nih.gov/health/advance-care-planning-healthcare-directives> | Over the 5-month timeframe, the number of user clicks on the UCHealth ACP patient portal page increased from 3511 in January to 6819 in April and 10,077 in May 2020. The total number of monthly ACP portal tool users increased from 418 in January to 1037 in April, then slightly decreased to 815 users in May. The weekly rate of Power of Attorney (POA) completions was 2.4-fold higher in the 9 weeks after 26 March 2020, compared to the 9 weeks prior, and this was the main use of the ACP portal (95.3%). The majority of users were female (67%), consistent with other evidence that males report less familiarity with concepts of ACP and POA. The mean age was 47.7 years (SD 16.1) and the largest age group of users were 25-34 years old - maybe because in Colorado, 33% of people diagnosed with COVID-19 were 20-39 years old, thus there was greater awareness about the importance of ACP even in the young and they are more likely to engage with an online platform. |
| Rabow MW, Keyssar JR, Long J, et al  2021  American journal of Hospice & Palliative medicine | Cohort study – retrospective  USA | The MERI Centre at UCSF, USA, provides primary palliative care education to health care teams | To outline the patient, family and professional education efforts of one palliative care education centre and detail the adjustments necessary and opportunities found in the COVID-19 crisis |  | The MERI centre runs a 2-part ACP workshop called "What matters most?". There was major growth in attendance during COVID-19. From April-December 2020, there were 125 participants (compared to 47 people in the 7 months prior to COVID-19).  Key lessons about palliative care education during a global pandemic;  1. Online learning has numerous advantages including allowing for increased participation and scale not possible with in-person learning, including national and international participants.  2. Intimacy and meaning are possible, and sometimes promoted, by the safety, convenience, and interactivity of online relationships.  3. The importance of palliative care education for frontline health care workers and for patients and their loved ones has been highlighted by the fear, disruption, and grief of the pandemic. |
| Valeri AM, Robbins-Juarez SY, Stevens JS, et al  2020  J Am Soc Nephrol | Cohort study – retrospective  USA | Patients with ESKD on dialysis admitted to a New York City hospital with COVID-19 | To examine presentation and outcomes of COVID-19 in patients with End stage kidney disease (ESKD) on dialysis |  | 59 patients were identified and the majority were on haemodialysis (n=57). Median age was 63 years, 56% were male and 75% were Hispanic ethnicity. 31% (n=18) of patients died in hospital - this group were older and had a higher Charlson comorbidity index. 14% (n=8) received mechanical ventilation - 75% of whom died. 19% (n=11) died with a "do not intubate" order.  The presentation of patients on dialysis with COVID-19 was similar to that of the general population. Patients with advanced age and co-morbidities are at the highest risk of severe COVID-19 manifestations. A high percentage of those who died had expressed wishes against intubation and mechanical ventilation, reflecting mandatory discussion and documentation of advanced directive in dialysis patients in America. |

**Supplementary Table 2: GUIDELINES**

| **Authors,  Year,  Journal** | **Country** | **Objectives** | **Resources (when available)** | **Main findings & comments** |
| --- | --- | --- | --- | --- |
| Farrell TW, Ferrante LE, Brown T, et al 2020  J Am Geriatr Soc | USA  Guidelines | To inform stakeholders about ethical considerations to consider when developing strategies for allocating scarce resources during COVID-19 involving older adults | <https://www.americangeriatrics.org/where-we-stand/covid-19> | Three key considerations are discussed;  1. Urgent need for ACP - ACP is important to reduce the need to ration limited health care resources during an emergency because these discussions will identify people who do not wish to receive intensive care, including mechanical ventilation.  2. Achieving justice in resource allocation - a preference for distributive justice that maximizes relevant clinical factors and either de-emphasizes or eliminates factors that place an arbitrary and disproportionate weight on advanced age.  3. Legal considerations - The position statement includes:  (a) avoiding age per se as a means for excluding anyone from care;  (b) assessing comorbidities and considering the disparate impact of social determinants of health;  (b) encouraging decision makers to focus primarily on potential short-term (not long-term) outcomes;  (d) avoiding ancillary criteria such as ―life-years saved and long-term predicted life expectancy that might disadvantage older people;  (e) forming and staffing triage committees tasked with allocating scarce resources;  (f) developing institutional resource allocation strategies that are transparent and applied uniformly;  (g) facilitating appropriate advance care planning |
| Janssen DJA, Ekstroem M, Currow DC, Johnson MJ, Maddocks M, Simonds AK, et al  2020  Eur Respir J | UK  Guidelines | To make consensus recommendations for palliative care for patients with COVID-19 | 90 international experts invited to complete an online survey including stating their agreement, or not, with 14 potential recommendations  ERS Guidelines  <https://www.ersnet.org/covid-19/covid-19-guidelines-and-recommendations-directory/> | 68 (75.6%) experts, from 15 countries and mostly physicians, responded in the first round and consensus (70% agreement) was achieved on 13 recommendations based upon indirect evidence and clinical experience.  Recommendations relevant to ACP include:  1) ACP should be routinely performed or reviewed by clinicians with patients and their loved ones at diagnosis of serious COVID-19.  2) ACP should be re-evaluated prior to discharge of recovered COVID-19 patients from hospital.  3) Staff taking care of patients with serious COVID-19 should receive training in online clinician– family communication (while using telephone or video conferencing).  Barriers to ACP; COVID-19 evolves rapidly resulting in lack of clarity on the patient’s condition preventing long-term planning, patients may be too ill or too anxious to participate in ACP, family are not physically present to participate in ACP. Healthcare professionals rely on in-person and non-verbal cues to facilitate difficult conversations and may be reluctant to discuss sensitive topics during phone or video consultations. Remote communication is more challenging for people with low literacy or few digital literacy skills, and people with sight or hearing impairment. |
| Kuzuya M, Aita K, Katayama Y, Katsuya T, et al  2020  Geriatr Gerontol Int | Japan  Guidelines | To make ethical suggestions aimed at achieving a society in which older patients can receive the best medical and long-term care they deserve, even during the COVID-19 pandemic. In particular, to consider the timing and importance of ACP implementation. | The Japan Geriatrics Society "Guidelines for the decision-making processes  in medical and long-term care for the elderly". [Cited 5 Aug  2020]. Available from URL:  <https://www.jpn-geriat-soc.or.jp/proposal/guideline.html> (in Japanese) | Recommendations relevant to ACP include:  1.2. ACP should be promoted to ensure the right to “best medical and long-term care” until the end-of-life.  1.3. The end-of-life care desired by older persons should be guaranteed.  2.1. Medical information needs to be shared with the patient and family, and active decision support needs to be provided.  2.2. It is necessary to ensure communication between the older person and family, and medical and long-term care workers. |

**Supplementary Table 3: REVIEWS**

| **Authors,  Year,  Journal** | **Country Study design** | **Objectives** | **Main findings & comments** |
| --- | --- | --- | --- |
| Farrell TW, Francis L, Brown T, et al  2020  J Am Geriatr Soc | USA  Narrative review | a companion manuscript to the American Geriatrics Society position statement (also by Farrell et al 2020), intended to inform stakeholders about ethical considerations that should be considered when developing strategies for allocation of scarce resources during COVID-19 involving older adults | Three key consideration as described above.  Value of individual choice and advance directives - respect for autonomy is an important moral consideration and patient choice should be honoured. In emergency situations like COVID-19, identification of the patient's surrogate decision maker is especially important.  It urges caution on 2 aspects of ACP during COVID-19;  1. Critically ill COVID-19 patients may not have ACP or be in a position to make their wishes known - in this case, they should not be pressured to make care decisions based on conserving resources.  2. Physicians should not engage in pre-emptive rationing, where pressure is placed on older adults to reconsider their ACP or elect for Do-not-resuscitate orders |
| Gilissen J, Pivodic L, Unroe KT, et al  2020  J Pain Symptom Manage | USA Narrative review | To identify, review, and compare national and international COVID-19 guidance for nursing homes concerning palliative care, issued by government bodies and professional associations | 21 eligible documents were identified. International documents focused primarily on infection prevention and control, including only a few sentences on palliative care related topics. Palliative care themes most frequently mentioned end-of-life visits, ACP documentation, and clinical decision making toward the end of life (focusing on hospital transfers).  In relation to ACP;  - 14 documents mention ACP primarily in relation to transfers to hospital and/or focused primarily on written plans or orders to guide emergency situations.  - 8 documents state that consulting a person’s ACP is crucial when deciding whether they should be hospitalized.  - A matter of urgency is acknowledged in all documents, stating that residents’ advance directives/advance care plans should be completed and up to date.  - In U.S. documents, it is added that written physician orders must reflect patients’ wishes.  - 7 documents are more specific to what the advance care plan should at least entail (e.g. Power of attorney, CPR, admission to hospital, intubation, non-invasive ventilation, fluids, antibiotics).  - 4 documents acknowledge that plans may need to be made in emergency situations, with little time available. - 6 documents highlight the importance of involving representatives/family in goal-setting or ACP.  - 3 documents explicitly state that ACP is a person-centred approach to care, that involves ‘‘effective’’ or ‘‘adequate, sympathetic’’ communication and a thorough understanding of a person’s life, values, priorities, and preferences.  Overall, ACP is mentioned in many documents but is discussed in a very limited way. The emphasis lies on treatment preferences in writing while the actual communication process is less frequently mentioned. |
| Gupta A, Bahl B, Rabadi S, Mebane A, 3rd, Levey R, Vasudevan V  2021  Am J Hosp Palliat Care | USA Narrative review | To describe the challenges and unique opportunities of ACP in the COVID-19 era. | Benefits of ACP; respect patient autonomy, lower costs to healthcare system due to reduction in aggressive treatment, leading to a potential reduction in resource competition during COVID-19.  Barriers to ACP;  a) Patient factors - poor uptake due to some reticence to discuss End of Life Care, patients not willing to raise ACP with clinicians, ACP uses difficult to understand language, the perception that ACP can be inconsistent with patient goals. The distribution of ACP is unequal across races – it is higher in the elderly, white population and critically ill, but lower in ethnic minorities with lower SES. This disparity is driven by experiences of discrimination and mistrust of HCW. Ethnic minorities are more likely to have appointed a Next of Kin, but less likely to have documented ACP, leading to increased stress on substitute decision makers.  b) Healthcare factors - clinicians may delay end of life conversations due to inaccurate prognosis, the focus on ACP in hospital & during critical illness delays difficult conversations until necessary, reluctance to take away patients’ hope of recovery. All stems from lack of ACP training for clinicians & paucity of palliative care medical education. Also varying rates of adherence to and implementation of ACP.  Suggestions to increase ACP; increase patient access to online health records to facilitate documentation of ACP, increase frequency of requests to complete ACP, take advantage of key occasions to complete ACP (e.g. admission to nursing home), increase educational & awareness campaigns. |
| Liao CT, Chang WT, Yu WL, et al  2020  Rev Cardiovasc Med | Taiwan Narrative review | To provide guidance for the management of critical cardiovascular disease during the COVID-19 pandemic | The article details management of patients with COVID-19 and; - acute coronary syndromes - cardiogenic shock - acute heart failure - CPR ACP should be prioritised and discussed early to avoid high-intensive care and non-beneficial treatments. Patient's goals and expectations should be discussed and shared with their proxies. |
| Salins N, Mani RK, Gursahani R, et al  2020  Indian J Crit Care Med | India Narrative review | To address the symptom management and supportive care strategies in patients with COVID-19 who are suitable for intensive care treatment and ventilation | 19% of COVID-19 patients develop severe illness requiring hospitalisations, and breathlessness is the most common symptom in patients needing ICU treatment.  The article includes details on management of COVID-19 patients suffering from:  - breathlessness  - delirium  - respiratory secretions  - pain  - intractable symptoms and end-of-life care  The suffering of patients with COVID-19 and their families is both physical and psychological, social and spiritual. As such, the demand for palliative care services is increased. It is important to discuss goals of care with patients and their families, to ascertain preferences about treatment and place of care. In particular, elderly patients with co-morbidities, organ failure, cancer and impairment who have COVID-19, should have the advantages and disadvantages of ventilation and ICU treatment explained. This is to avoid ICU admissions in COVID-19 patients who are unlikely to benefit. Early ACP also spares patients and families from complex triage discussions. |
| Selman L, Lapwood S, Jones N, Pocock L, Anderson R, Pilbeam C, et al  2020  The Centre for Evidence-Based Medicine | UK  Narrative review | To synthesise recent evidence regarding ACP in community settings, considering its implications with respect to COVID-19, and highlight existing guidelines and resources. | 203 records were screened and 21 research studies and 10 systematic reviews met inclusion criteria. 12 guidelines also identified.  Barriers to ACP in light of COVD-19;  a) Individual level - lack of consumer knowledge about ACP, perceived irrelevance of ACP, lack of trust and cultural reluctance to discuss death, speed of deterioration not allowing time to discuss ACP.  b) Interpersonal level - role ambiguity (Doctor and patient expectations that the other will initiate ACP discussion), social distancing prohibiting face-to-face ACP conversations, family shock and grief not conducive to discussing ACP.  c) Clinician level - lack of clinician knowledge, skill, confidence, time to initiate ACP and concern it will deprive patients of hope and create anxiety.  d) System level - lack of linkages and mechanism for sharing ACP, lack of funding, lack of standardisation of templates, tool, IT systems.  Enablers to ACP in light of COVID-19; increased public awareness and education on ACP, COVID-19 provides a trigger to initiate ACP discussion, increased attention on IT systems, development of new templates and tools to facilitate ACP, uptake of video and tele-health facilitating remote ACP, unique opportunity to create integrated web-based systems for ACP linked to electronic medical records |
| Selman LE, Chao D, Sowden R, Marshall S, Chamberlain C, Koffman J  2020  Journal of Pain & Symptom Management | UK  Narrative review | To review bereavement risk factors in COVID-19 and provide evidence-based recommendations for how to support bereaved relatives. | Bereavement risk factors in COVID-19 include patient isolation, limited family access, virtual communication and lack of ACP. In particular, relevant to ACP;  - If no ACP is documented, a patient's values and preferences for high-intensity treatments cannot be known, and leaves family members ill-prepared for their death.  - Personal protective equipment depersonalises and complicates face-to-face ACP communication, especially for the hearing impaired and frail. |
| Vipperman A, Zimmerman S and Sloane PD  2021 J Am Med Dir Assoc | USA Narrative review | To summarize recommendations issued by key organizations related to COVID-19 in Assisted Living Communities, focussing in healthcare and quality of life. | Recommendations on ACP:  - Staff should receive training on ACP and serve as a conduit for primary care providers, to have these discussions with residents - The resident's goals of care should be considered when making transfer decisions in relation to COVID-19. |
| West E, Moore K, Kupeli N, Sampson EL, Nair P, Aker N, et al  2021  Age Ageing | UK  Narrative review | To explore key factors that influence Place of care and Place of death decisions in older adults and to investigate key factors that influence the process and outcome of these decisions in older adults | 533 papers were identified and 10 papers met inclusion criteria. In terms of ACP, it is a welcomed process in nursing homes and community settings. There is limited evidence that ACP support proxies to make consistent healthcare decisions. The evidence showing good accord between patient and proxy decisions is lacking. ACP engagement and comprehension levels differ based on income level, patient attitudes, comfort with and trust in the healthcare system.Care should be taken to ensure that access to ACP services and interventions is culturally appropriate and based on patient values. In the COVID-19 context, ACP needs to be continually renegotiated and reconsidered as one's health status and care options change. |
| Bolt SR, van der Steen JT, Mujezinović I, et al  2021  Int J Nurs Stud | Netherlands Rapid review | To formulate practice recommendations for nursing staff with regard to providing palliative dementia care in times of COVID-19 | 23 documents were included. Articles specifically addressing palliative care in dementia in relation to COVID-19 were limited. Recommendations relevant to ACP include;  1. Nursing and medical staff should collaborate to review (existing) ACP.  2. Ideally ACP before a diagnosis of COVID-19 and before the person with dementia loses cognitive capacity. Provide patients and families with information and guidance to facilitate ACP. Consider utilising decision aids to support families.  3. Facilitate timely ACP by introducing the COVID-19 subject and discuss potential scenarios and end-of-life care options.  4. Be aware that ACP conversations may be coloured by fears or emotions induced by the COVID-19 pandemic.  5. Consider using ACP conversations to identify anticipatory grief and to provide information on bereavement preparation in times of COVID-19.  6. Make sure to document wishes clearly in transferable (digital) files that should be available and accessible at all times for different care agencies and care personnel.  7. During this pandemic, nursing staff may discuss the preferred place of death with persons with dementia, before potential infection. Keep in mind that a familiar environment is likely preferred over a hospital.  8. Complex decisions about hospitalization related to COVID-19 should be discussed in a multi-disciplinary team (MDT).  9. For people with dementia who have COVID-19, discuss goals of care with the patient and MDT as soon as possible and revise care goals as the situation changes.  10. Be sensitive to family members and proxy decision-makers, who may have to make hasty, difficult and emotive decisions on behalf of their relative. |
| McMahan RD, Tellez I, Sudore RL  2021  J Am Geriatr Soc | USA  Scoping review | To describe the existing ACP landscape, conduct a scoping review of recent randomized trials in the past decade to describe current ACP interventions and outcomes, and provide recommendations for future research | Of 1464 articles identified, 69 Randomised Control Trials met eligibility and 94% were rated high quality. The ACP definitions, age criteria, diseases and settings varied. The interventions medium was also varied, and included discussions, video, interactive multimedia, written form and clinician training. Outcomes for all ACP interventions were predominantly positive. |

CASE SERIES/CROSS-SECTIONAL

| **Authors,  Year,  Journal** | **Country Study design** | **Objectives** | **Population and setting** | **Main findings & comments** |
| --- | --- | --- | --- | --- |
| Biswas S, Adhikari SD, Bhatnagar S  2020  Indian J Palliat Care | India  Case series | To share the experience of three patients with advanced cancer where telemedicine was used for the process of providing End-of-life care. | Palliative care services in India, in particular the cases of 3 patients with advanced cancer are discussed | Case 1; 68 year old male with metastatic lung cancer. His main symptom was dyspnoea. Telemedicine used to assess symptoms and advise on management, including titration of opioids and use of fan. Psychological support provided to carers and ACP discussed.  Case 2; 45 year old female with metastatic cholangiocarcinoma. Telemedicine used to identify terminal breathing. ACP discussed with family.  Case 3; 52 year old female with metastatic gallbladder carcinoma. Telemedicine used to assess discomfort and restlessness. Terminal event identified and managed with family.  Telemedicine during COVID-19 enables connection with dying patients in remote locations, facilitates shared-decision making and ACP. It allows carers to be guided in symptom management, as well as providing psychological support to bereaved families. |
| Kotze C, Roos JL 2020 S Afr Fam Pract | South Africa Case report | To highlight the complexity of capacity assessments and ACP in people with serious mental illness (SMI) | 72 year old female with treatment resistant schizophrenia and terminal cancer | The case discusses the difficulties of assessing end-of-life decision-making capacity in people with SMI, using this patient as an example. Older people with SMI are able to engage in ACP and have decision-making capacity about their end-of-life care preferences, however people with SMI are significantly less likely to access palliative care services. More research about palliative care and ACP for people with SMI is needed and is urgent in light of the COVID-19 pandemic. |
| Rivet EB, Blades CE, Hutson M, et al  2020  Am Surg | USA  Case report | To highlight the elements of high-quality ACP in the surgical context | 74-year-old female with multiple co-morbidities who had ACP discussions in the context of proposed surgery for colon cancer | The patient was diagnosed with colon cancer, and noted to have multiple medical conditions and decreased functional status, as well as concerns for her decision-making capacity. Surgery was delayed due to COVID-19 pandemic. An ACP conversation was had prior to the planned surgery which later formed the basis of her healthcare proxy (HCP) to limit interventions (like CPR and intubation) when the patient contracted COVID-19.  Key points included;  1. For best outcomes, ACP conversations should occur before a crisis, allowing patients to understand ACP options and reflect on personal values and care preferences. ACP should be revisited over time as circumstances change.  2. ACP conversations should occur whilst the patient has capacity.  3. Including HCPs in ACP discussion is beneficial for the patient, proxy and health care team.  4. Skillful ACP conversations help patients to distinguish between desired and undesired outcomes and can define a patient's acceptable levels of function, allowing better definition of treatment limitations.  5. The meaning of quality-of-life statements vary considerably between patients.  6. Individuals who request full active treatment are usually able to describe a point at which they would be willing to accept a natural death. Having individuals reflect on potential outcomes might help them articulate their goals and the circumstances under which they would want to limit treatment.  7. Elective surgical intervention provides an opportunity for ACP conversations, by engaging patients in discussions about the goals of care, and potential avoids undesired care. |
